# Work related complaints of neck, shoulder and arm among computer office workers: a cross-sectional evaluation of prevalence and risk factors in a developing country

**DOI:** 10.1186/1476-069X-10-70

**Published:** 2011-08-04

**Authors:** Priyanga Ranasinghe, Yashasvi S Perera, Dilusha A Lamabadusuriya, Supun Kulatunga, Naveen Jayawardana, Senaka Rajapakse, Prasad Katulanda

**Affiliations:** 1 Diabetes Research Unit, Department of Clinical Medicine, Faculty of Medicine, University of Colombo, Colombo, Sri Lanka; 2 Department of Clinical Medicine, Faculty of Medicine, University of Colombo, Colombo, Sri Lanka

**Keywords:** Computer workers, Ergonomics, Prevalence, Risk factors, Musculoskeletal diseases

## Abstract

**Background:**

Complaints of arms, neck and shoulders (CANS) is common among computer office workers. We evaluated an aetiological model with physical/psychosocial risk-factors.

**Methods:**

We invited 2,500 computer office workers for the study. Data on prevalence and risk-factors of CANS were collected by validated Maastricht-Upper-extremity-Questionnaire. Workstations were evaluated by Occupational Safety and Health Administration (OSHA) Visual-Display-Terminal workstation-checklist. Participants' knowledge and awareness was evaluated by a set of expert-validated questions. A binary logistic regression analysis investigated relationships/correlations between risk-factors and symptoms.

**Results:**

Sample size was 2,210. Mean age 30.8 ± 8.1 years, 50.8% were males. The 1-year prevalence of CANS was 56.9%, commonest region of complaint was forearm/hand (42.6%), followed by neck (36.7%) and shoulder/arm (32.0%). In those with CANS, 22.7% had taken treatment from a health care professional, only in 1.1% seeking medical advice an occupation-related injury had been suspected/diagnosed. In addition 9.3% reported CANS-related absenteeism from work, while 15.4% reported CANS causing disruption of normal activities. A majority of evaluated workstations in all participants (88.4%,) and in those with CANS (91.9%) had OSHA non-compliant workstations. In the binary logistic regression analyses female gender, daily computer usage, incorrect body posture, bad work-habits, work overload, poor social support and poor ergonomic knowledge were associated with CANS and its' severity In a multiple logistic regression analysis controlling for age, gender and duration of occupation, incorrect body posture, bad work-habits and daily computer usage were significant independent predictors of CANS

**Conclusions:**

The prevalence of work-related CANS among computer office workers in Sri Lanka, a developing, South Asian country is high and comparable to prevalence in developed countries. Work-related physical factors, psychosocial factors and lack of awareness were all important associations of CANS and effective preventive strategies need to address all three areas.

## Background

Complaints of arms, neck and shoulders (CANS) is defined as the presence of musculoskeletal complaints of the said region not caused by acute trauma or by any systemic disease [1]. CANS is common among computer office workers worldwide and is a well-recognized cause of occupational illness leading to frequent absenteeism from work, reduction in overall productivity, poor quality of life and escalating medical expenses [2,3]. In the United States, CANS is a leading cause of occupational illness with annual costs related to absenteeism from work and treatment being $45-54 billion [4]. The recent increase in computer-related work as a consequence of rapid industrialization has considerably increased the prevalence of CANS among computer office workers not only in western developed countries but also in developing countries such as Sudan and Sri Lanka [5,6].

The aetiology of CANS among computer office workers is complex and poorly defined. Recently several studies have defined and identified potential risk factors for CANS, such as physical exposure resulting from static body postures, repetitive tasks and workplace design [7,8]. In addition, psychosocial factors such as high quantitative job demands, minimal autonomy and limited peer support have also been implicated [9,10]. Thus, it is important that an aetiological model for CANS, consider both physical and psychosocial factors. A significant majority of risk factor studies are from western developed countries and at present there are no studies from developing countries in the South-Asian region. Sri Lanka is a rapidly developing nation in South Asia having a population of about 19 million people [11]. Computer systems are being increasingly utilized to support the rapid industrial development, while ten percent of Sri Lankan households are known to be using personal computers [12]. The estimated one-year prevalence of CANS in Sri Lanka is 63.6% [6], which is comparable to the prevalence in developed countries. Hence, CANS among computer office workers in Sri Lanka is likely to be causing a significant personal, industrial and economical impact. However, in order to design preventive strategies, identification of high risk sub-groups and an aetiological model needs to be defined with the recognition and quantification of risk factors and their interactions.

The Musculoskeletal Upper Extremity Questionnaire (MUEQ) is a validated tool used to assess the occurrence and nature of CANS and work-related physical and psychological risk factors [13]. The translated version of the MUEQ has shown satisfactory psychometric properties for it to be used to assess work-related risk factors for development of CANS among Sri Lankan computer office workers [6]. The psychosocial risks factors measured in the MUEQ are derived from the "Karasek" model [14,15]. The basis of this model is that psychological strain does not result not from a single aspect of the work environment but from a combined effect of the level of; job demands, autonomy and social support [14-17]. The present study aims to analyze the presence of CANS in relation to the effects of exposures to physical and psychological factors, and their probable interactions. In addition we aim to analyse the effects of workstation design on the presence of CANS and study the knowledge and awareness pertaining to ergonomical practices among computer office workers in Sri Lanka.

## Methods

### Study participants and Sampling

The present study was conducted between May and December 2009. Two thousand five hundred computer office workers from two telecommunications institutes and a computer training institute with branches in all of the nine provinces of Sri Lanka were invited for the study [18]. The number of participants to be invited from each province was determined by the probability proportionate to sample size (PPS) method depending on the percentage of computer users in each province as determined by the Department of Census and Statistics, Sri Lanka [12]. Informed written consent was obtained from each study participant. To be included, an office worker had to be employed in the current position for at least twelve months and use computers to complete their job tasks for at least two hours per day. Participants were excluded based on the following criteria: (1) suffering from diseases affecting the muscluskeletal system such as Rheumathoid Arthritis, Osteoarthritis and other Connective Tissue Disorders; (2) having a previous surgery of the upper musculoskeletal extremity. A list of employees satisfying the inclusion criteria was obtained from the human resources department of the respective institutes and they were screened for the presence of exclusion criteria. This final list of employees was subjected to simple random sampling by computer generate numbers and the selected employees were invited for the study. Ethical approval for the study was obtained from the Ethics Review Committee of the Faculty of Medicine, University of Colombo, Sri Lanka.

### Study instruments

Data on prevalence of CANS and its potential work-related physical and psychological risk factors were collected by means of the validated Maastricht Upper extremity Questionnaire (MUEQ) [6]. The MUEQ has been translated and validated for the Sri Lankan population, and has shown satisfactorily psychometric properties (i.e. factor structure and internal consistency) which has been previously reported [6]. The MUEQ evaluated risk factors in six different domains (scales); Workstation, Body posture, Job control, Job demand, Break time and Social support. Each scale was further subdivided into two sub-scales (factors). Factor structure, internal consistency and item total correlations have been discussed elsewhere [6]. The two sub-scales in the Workstation domain were work area (3 items, 0-6 points) and computer position (3 items, 0-6 points). In the body posture scale, incorrect body posture (4 items, 0-20 points) and bad work-habits (5 items, 0-25 points) were the two sub-scales. Skill and abilities (4 items, 0-20 points) and decision making (4 items, 0-20 points) were the two sub-scales in the Job control domain, while in the Job demand domain time management (4 items, 0-20 points) and work overload (3 items, 0-15 points) were the sub-scales. The break time domain consisted of work breaks (3 items, 0-15 points) and variations in work (3 items, 0-15 points) as the two sub-scales. Finally, the social support domain contained the two sub-scales work environment (3 items, 0-15 points) and social support (3 items, 0-15 points). Age, sex and previous history of complaints were regarded as potential confounders and were considered as independent risk factors of CANS. The outcome variable was the occurrence of complaints of the; a) neck, b) shoulder and arm, and c) forearm and hands (the questions were asked for each region separately) with a duration of at least one week during the preceding 12 months. The risk factor analysis was conducted for each area independently.

Individual workstations were evaluated by the validated Occupational Safety and Health Administration (OSHA) VDT workstation checklist [19]. The OSHA VDT workstation checklist is used specifically for identifying risk factors for work-related CANS associated with workstation postures and devices. The 33 items in the OSHA VDT workstation checklist identifies specific risks for work-related symptoms related to positioning of the head, neck, shoulders, and trunk, as well as seating issues. The VDT workstation checklist also identifies risks associated with keyboard and mouse position, monitor position, and lack of document holder, arm and wrist rests, and telephone hands-free headset.

The participant knowledge and awareness of ergonomics, and the extent to which the principles of ergonomics were put into practice in the work-place were evaluated by using a set of expert-validated self-administered questions. Ten pictorial questions evaluated participants' knowledge on correct postures and equipment placement, each correct answer was given one mark (total score-10).

### Statistical Methods

A binary logistic regression analysis was performed to investigate the association of risk factors separately for a) neck, b) shoulder and arm and c) forearm and hand complaints. In addition a similar binary logistic regression analysis (0-Mild, 1-Severe) was performed in those with CANS to evaluate risk factors determining severity, severe cases were defined as those subjects who reported complaints in more than one of the body regions during while the pain was chronic (lasting for over a month) and present even after a short rest. The 'explained variance' of each of the logistic regression models was calculated by means of Nagelkerke's R^2 ^and the goodness of fit by means of the Hosmer and Lemeshow goodness-of fit test. A multiple logistic regression was conducted using presence of CANS as the categorical dependant variable (0 = No, 1 = Yes), using the independent variables identified as risk-factors in the binary logistic regression analysis of each region, while controlling for potential confounders such as age, gender and duration of occupation. All data were double entered and cross checked for consistency. Data were analyzed using SPSS version 14 (SPSS Inc., Chicago, IL, USA) statistical software package. A p-value ≤ 0.05 was considered statistically significant.

## Results

### Demographic characteristics

Sample size was 2210 (response rate - 88.4%). Mean age was 30.8 ± 8.1 years (range 18-60 years) and 50.8% were males. A majority (48.1%) of the study population was aged between 20-29 years, 46.5% males and 49.6% females belonged to this age group. A significant majority (87.4%) of the study population was right-handed (Males-84.7%; Females-90.3%). Seventy five percent of the study population had worked between 1-5 years in their current position. Of the male participants, 45.6% worked 6-9 hours per day with a computer, compared to 42.8% of the female participants and 44.3% of the entire study population. Sample characteristics are summarized in Table 1.

**Table 1:** Characteristics of the study population

	All	Males	Females
Province (number required*)			

Western (1100)	1116 (50.5%)	548 (48.8%)	568 (52.2%)

Central (240)	280 (12.7%)	92 (8.2%)	188 (17.3%)

Sabaragamuwa (140)	170 (7.7%)	90 (8.1%)	80 (7.4%)

North-Western (160)	164 (7.4%)	80 (7.1%)	84 (7.7%)

Southern (160)	162 (7.3%)	82 (7.3%)	80 (7.4%)

Eastern (80)	84 (3.8%)	76 (6.8%)	8 (0.7%)

Northern (70)	84 (3.8%)	76 (6.8%)	8 (0.7%)

North-Central (80)	82 (3.7%)	42 (3.7%)	40 (3.7%)

Uva (60)	68 (3.1%)	36 (3.2%)	32 (2.9%)

Age			

< 20 years	100 (4.5%)	26 (2.3%)	74 (6.8%)

20 - 29 years	1062 (48.0%)	522 (46.5%)	540 (49.6%)

30 - 39 years	740 (33.5%)	400 (35.7%)	340 (31.2%)

40 - 49 years	196 (8.9%)	104 (9.3%)	92 (8.5%)

= 50 years	112 (5.1%)	70 (6.2%)	42 (3.9%)

Number of working years in current position			

1 to 5 years	1670 (75.6%)	846 (75.4%)	824 (75.7%)

6 to 10 years	210 (9.5%)	116 (10.3%)	94 (8.6%)

11 to 15 years	192 (8.7%)	84 (7.5%)	108 (10.0%)

15 years and more	138 (6.2%)	76 (6.8%)	62 (5.7%)

Number of working hours with computer/day			

2 to 5 hrs	532 (24.1%)	230 (20.5%)	302 (27.8%)

6 to 9 hrs	978 (44.2%)	512 (45.6%)	466 (42.8%)

> 9 hrs	700 (31.7%)	380 (33.9%)	320 (29.4%)

* Number required from each province, based on percentage computer usage in each province and total provincial population (PPS method)

### Prevalence of CANS

The 1-year prevalence of CANS in the study population was 56.9%. Prevalence of CANS in males and females were 54.7% and 59.2% respectively (p > 0.05). The most commonly reported complaints were in the forearm and hand region (42.6%), followed by neck complaints (36.7%) and shoulder and arm complaints (32.0%). The 1-year prevalence of complaints of the various upper extremity body regions were greater for females than for males (Table 2), however this difference was statistically significant only for the shoulder and arm complaints (p < 0.001). The prevalence of CANS was most in the Sabaragamuwa province (70.6%), followed by Uva (69.6%), Northern (66.7%), Eastern (64.7%), North-Western province (63.4%), North-Central (58.5%), Western (57.7%), Central (51.4%), and Southern (23.5%) provinces. The prevalence in Southern province was significantly lower than in the other provinces (p < 0.001).

**Table 2:** One year prevalence of CANS lasting for at least one week during the previous year

	N	All Prevalence (95% CI) (n = 2210)	Males Prevalence (95% CI) (n = 1122)	Females Prevalence (95% CI) (n = 1088)	p value*
Region					

Neck	812	0.37 (0.35 to 0.39)	0.35 (0.32 to 0.38)	0.38 (0.35 to 0.41)	NS

Shoulder & arm	708	0.32 (0.30 to 0.34)	0.28 (0.25 to 0.30)	0.37 (0.34 to 0.40)	<0.001

Forearm & hands	942	0.43 (0.41 to 0.45)	0.42 (0.39 to 0.45)	0.43 (0.40 to 0.46)	NS

Severity					

Mild cases	854	0.39 (0.37 to 0.41)	0.35 (0.33 to 0.38)	0.42 (0.39 to 0.45)	<0.01

Severe cases	404	0.18 (0.17 to 0.20)	0.19 (0.17 to 0.22)	0.17 (0.15 to 0.20)	NS

Neck					

Right	188	0.08 (0.07 to 0.10)	0.08 (0.07 to 0.10)	0.09 (0.07 to 0.11)	NS

Left	78	0.03 (0.02 to 0.04)	0.02 (0.01 to 0.03)	0.05 (0.04 to 0.07)	<0.001

Both	546	0.25 (0.23 to 0.26)	0.25 (0.23 to 0.28)	0.24 (0.22 to 0.27)	NS

Shoulder and arm					

Right	142	0.06 (0.05 to 0.08)	0.07 (0.05 to 0.08)	0.06 (0.05 to 0.08)	NS

Left	125	0.06 (0.05 to 0.07)	0.04 (0.03 to 0.06)	0.07 (0.05 to 0.08)	<0.05

Both	441	0.20 (0.18 to 0.22)	0.16 (0.14 to 0.19)	0.24 (0.21 to 0.26)	<0.001

Forearm and hands					

Right	336	0.15 (0.14 to 0.17)	0.17 (0.15 to 0.20)	0.13 (0.11 to 0.15)	<0.01

Left	88	0.04 (0.03 to 0.05)	0.04 (0.03 to 0.05)	0.04 (0.03 to 0.05)	NS

Both	518	0.23 (0.22 to 0.25)	0.21 (0.19 to 0.24)	0.26 (0.23 to 0.29)	<0.01

*p value for Males vs. Females, NS - not significant, N-Number of subjects with complaints

Participants who reported complaints in the upper extremity were classified into two sub-groups: (1) mild cases: subjects who reported complaints in only one of the body regions; (2) severe cases: subjects who reported complaints in more than one of the body regions while the pain was chronic (lasting for over a month) and present even after a short rest. A majority of subjects of study participants reporting CANS had mild symptoms (67.9%), while only 32.1% of those with CANS complained of severe symptoms. The prevalence of mild and severe symptoms in all participants, males and females are presented in Table 2. In both males and females complaints of the "right side" were reported more frequently than for the "left side", however in both genders bi-lateral complaints were significantly more prevalent than unilateral complaints (Table 2). The common symptoms of the upper musculoskeletal extremity in those with complaints were pain (67.1%), fatigue and exhaustion (45.0%), stiffness (44.0%), numbness and tingling sensation (26.9%) and weakness (22.7%).

In study participants with CANS, 15.7% (n = 348) had taken treatment from a health care professional for his/her ailments, the treatment given was analgesic medication in 34.5%, physiotherapy in 28.3% and offering of surgical measures in 3.7%. However, only in 4 (1.1%) study participants seeking medical advice an occupation related injury had been suspected by the health care professional with the institution of necessary prevention strategies. In addition 9.3% (n = 206), reported CANS-related absenteeism from work, while 15.4% (n = 340) reported CANS causing disruption of their normal activities (Work-20.6%, Leisure-24.1% and both 55.3%).

### Workstation evaluation

A total of 2210 VDT workstations were evaluated by using the OSHA VDT workstation checklist. A significant majority of the workstations evaluated (88.4%, n = 1954) were non-compliant with the OSHA VDT workstation checklist. In those with non-compliant workstations (n = 1954) CANS were present in 59.2%. In addition in the 1258 study participants who suffered from one or more complaints of the upper musculoskeletal extremity, 91.9% had non-compliant workstations. The main deficiencies identified in workstation design were; the difficulties in performing computer tasks and telephone use at the same time (58.4%), difficulty in keeping upper arm and elbow close to the body while working (45.8%), Shoulders and arm being in awkward positions during work (40.6%), improper placement of document holder (40.4%) and non-supportive or lack of arm rests (39.9%) (Table 3).

**Table 3:** Deficiencies identified with the OSHA VDT workstation checklist

Deficiency in VDT workstation design	Percentage N = 1954	Item number*
Difficulty in performing computer & telephone tasks at same time	58.4%	31

Difficulty in keeping upper arms and elbow close to body	45.8%	5

Awkward position of shoulders and arms	40.6%	4

Improper placement of document holder	40.4%	28

Non-supportive armrest	39.9%	15

Poor design of wrist rest	35.4%	29

Seat front pressing against back of knees (seat design)	35.2%	13

Difficulty in keeping wrists and hands straight	34.9%	7

Improper placement of wrist rest	32.8%	30

Foot not resting on ground	32.0%	9

Thighs not being parallel	30.4%	8

Difficult in keeping forearms and wrists straight	29.9%	6

Wrist and hands resting on sharp/hard edge	29.6%	19

Poor maintenance of equipment	29.4%	33

Seat cushion poorly designed	28.8%	14

Equipment not adjustable to suit requirement	28.8%	32

Non-supportive backrest	27.8%	11

Difficulty in keeping trunk perpendicular	27.6%	3

Monitor distance being too far	26.6%	22

Input devices (Keyboard and mouse) placed improperly	26.5%	17

Head, neck and trunk in a twisted position while working	26.4%	2

Glare present on screen	23.6%	24

Computer tasks not varied with insufficient breaks	22.9%	10

Top-line of screen being above eye level	22.8%	20

Head and neck bent while working	20.6%	1

No clearance space underneath the table	20.4%	26

Insufficient seat width and length	18.7%	12

Monitor placement not directly in front	18.4%	23

Difficulty in working while using spectacles	17.3%	21

Thighs being trapped under the computer table	16.0%	25

Non-stable input device (Keyboard and mouse) platform	15.6%	16

Non-stable document holders	12.6%	27

Non-suitable type of input device	11.3%	18

* Item number in OSHA VDT workstation checklist (Appendix 1)

### Potential Risk Factors of CANS

According to the binary logistic regression analysis analyses female gender, daily computer usage, incorrect computer positioning, incorrect body posture, bad work-habits, work overload and poor social support, were significantly associated with neck complaints (Table 4). The Nagelkerke's R^2 ^was 0.36 and the Hosmer-Lemeshow goodness-of-fit test was not significant (χ^2 ^= 5.41, p = 0.700). The binary logistic regression analyses showed a significant association between shoulder complaints and female gender, daily computer usage, incorrect body posture, lack of autonomy, work overload and poor social support (Table 4). The Nagelkerke's R^2 ^was 0.42 and the Hosmer-Lemeshow goodness-of-fit test was not significant (χ^2 ^= 3.32, p = 0.664).

**Table 4:** Psychological and physical risk factors determining complaints of each region (neck, shoulder/arms, forearm/hand) and the severity of complaints

Risk factors	Odds ratio (95% CI)			
	
	Neck	Shoulders & arms	Forearm & hands	Severe complaints
Age	0.99 (0.97-1.01)	0.97 (0.96-1.00)	0.96 (0.94-0.98)	0.96 (0.93-0.98)

Female gender	1.26 (1.03-1.39)*	1.59 (1.31-1.88)*	1.33 (1.11-1.56)*	1.17 (1.10-1.24)*

Duration of occupation	1.03 (1.01-1.05)	1.00 (0.98-1.02)	1.06 (1.03-1.09)	0.98 (0.95-1.02)

Daily computer usage	1.13 (0.99-1.27)*	1.98 (1.46-2.51)*	1.18 (1.06-1.30)*	1.92 (1.88-1.97)*

Work area	0.94 (0.88-0.99)	0.93 (0.88-0.98)	0.96 (0.91-1.01)	0.82 (0.74-0.89)

Computer positioning	1.15 (1.08-1.23)*	1.01 (0.95-1.07)	0.97 (0.92-1.03)	1.08 (0.99-1.18)

Incorrect body posture	1.36 (1.13-1.48)*	1.23 (1.11-1.36)*	1.15 (1.03-1.26)*	1.59 (1.55-1.63)*

Bad work-habits	1.18 (1.13-1.23)*	1.05 (1.02-1.08)	1.10 (1.06-1.14)*	1.11 (1.06-1.15)*

Skills and abilities	1.01 (0.98-1.04)	0.99 (0.96-1.02)	0.99 (0.97-1.03)	1.09 (1.04-1.14)

Decision making	0.98 (0.95-1.01)	1.13 (1.08-1.18)*	1.17 (1.14-1.20)*	1.16 (1.12-1.21)*

Time management	0.95 (0.92-0.98)	0.92 (0.89-0.95)	0.97 (0.95-1.00)	1.05 (1.00-1.10)

Work overload	1.11 (1.07-1.15)*	1.18 (1.14-1.22)*	1.29 (1.16-1.41)*	1.56 (1.51-1.60)*

Work breaks	0.90 (0.86-0.94)	1.03 (0.98-1.08)	1.04 (0.99-1.09)	0.92 (0.86-0.98)

Variation in work	1.01 (0.97-1.05)	1.09 (1.05-1.13)	1.01 (0.97-1.04)	1.17 (1.10-1.24)

Work environment	1.02 (0.97-1.08)	0.87 (0.82-0.92)	0.99 (0.94-1.05)	0.88 (0.81-0.96)

Social support	1.14 (1.09-1.19)*	1.17 (1.12-1.22)*	1.09 (1.06-1.12)*	1.23 (1.15-1.33)*

Ergonomic knowledge	1.08 (1.06-1.10)*	1.01 (0.96-1.06)	1.10 (1.05-1.15)*	1.03(0.97-1.09)

* p < 0.05

The binary logistic regression analyses showed significant associations between forearm/hands complaints and female gender, daily computer usage, incorrect body posture, bad work-habits, lack of autonomy, work overload and poor social support (Table 4). The Nagelkerke's R^2 ^was 0.42 and the Hosmer-Lemeshow goodness-of-fit test was not significant (χ^2 ^= 5.26, P = 0.412). Female gender, daily computer usage, incorrect body postures, bad work-habits, lack of autonomy, work overload and poor social support were all significantly associated with the presence of severe CANS in the binary logistic regression analysis (Table 4). The Nagelkerke's R^2 ^was 0.39 and the Hosmer-Lemeshow goodness-of-fit test was not significant (χ^2 ^= 5.43 P = 0.624).

In multiple logistic regression, while controlling for age, gender and duration of occupation, the incorrect body posture (OR-1.170 [1.141-1.201]), bad work-habits (OR- 1.231 [1.204-1.261]), daily computer usage (OR-1.269 [1.263-1.275]), work overload (OR- 1.117 [1.110-1.123]) and poor social support (OR-1.089 [1.080-1.098]) were significant independent predictors of presence of neck complaints (p < 0.001). In a similar analysis, incorrect body posture (OR-1.350 [1.320-1.380]), bad work-habits (OR- 1.323 [1.303-1.343]) and daily computer usage (OR-1.119 [1.083-1.157]) were significant independent predictors of presence of shoulder and arm complaints (p < 0.001). Similarly, for forearm and hand complaints, incorrect body posture (OR-1.187 [1.157-1.207]), bad work-habits (OR- 1.210 [1.204-1.217]), daily computer usage (OR-1.109 [1.083-1.134]) and poor social support (OR-1.106 [1.086-1.127]) were the only significant independent predictors of presence of CANS (p < 0.001).

### Knowledge and awareness of ergonomics

A majority of study participants were not aware about the term 'Ergonomics' (70.1%), while only 14.0% defined the term correctly. In those who have ever heard of the term 'Ergonomics', 39.1% had heard of it at the present workplace/institute, 32.5% via the internet, 16.6% via media (television/radio/newspapers), 14.6% at a workshop or conference, 14.2% from colleagues and 3.6% from a health care professional. In those who had heard of the term 'Ergonomics' (29.9%, n = 660), only 44.6% (n = 295) said that they were aware about the correct postures/equipment placement and implemented them at the workplace. The commonest reasons for non-implementation were; the lack of proper facilities (34.6%) and being not convinced of the impact (25.5%). The mean score for the ten pictorial questions were 5.8 ± 2.3 (range 0-9). The mean score in those with and without CANS were 4.7 ± 1.8 and 6.9 ± 1.1 respectively (p < 0.001). Poor ergonomic knowledge was also a significant predictor for neck and, forearm and hand complaints (Table 4).

## Discussion

The present study is the first comprehensive report on the prevalence and risk factors of CANS among computer office workers from a developing South-Asian county. The observed prevalence of CANS among the Sri Lankan computer office workers was 56.9%, we also found that the reported complaints in the forearm and hand region was much higher than neck and shoulder complaints. The observed prevalence was similar to the prevalence reported from other developed countries (Table 5) [5,13,20-22]. However, there is a scarcity of data from other similar developing countries in the region. In addition, we also demonstrate that CANS in Sri Lanka has a potential to compromise workers quality-of-life and increase medical expenses as 22.7% had taken treatment from health care professionals for their ailments, while 9.3% reported CANS-related absenteeism from work, and 15.4% reported CANS causing disruption of their normal activities.

**Table 5:** Prevalence of CANS among computer office workers worldwide

Country		Prevalence %		
	
	All regions	Neck	Shoulder and arm	Forearm and hand
Sri Lanka	56.9	36.7	32.0	42.6

Sudan [6]	53.0	64.0	32.0 - 41.0	19.0 - 29.0

Netherlands [14]	54.0	33.0	31.0	7.0 - 11.0

Greece [21]	64.0	55.8	23.5- 40.0	39.8

Finland [22]	--	63.0	24.0	35.0

Denmark [23]	--	37.2	--	21.5

Upper musculoskeletal extremity complaints among computer office workers are known to be associated with both work-related psychosocial and physical factors [2,4,23,24]. The present study shows that among the work related physical factors irregular body posture at work (twisted head and body, bent head and asymmetrical trunk) and bad work habits (sitting for long hours in one position, working with lifted shoulders and performing repetitive tasks) were significantly associated with CANS. These factors were also determinants of the severity of CANS among the study population, suggesting a dose-response relationship [25,26]. However, the present study is not a prospective study and it is also possible that these factors could have exacerbated non-work related symptoms. Increasing hours of daily computer usage was also consistently associated with complaints in all regions and severity. In addition a majority of the workstations in the present study were ergonomically poorly designed and symptoms were more prevalent in those with poorly designed workstations. In the scientific literature there is consensus that poor ergonomic conditions at workstations contributes to musculoskeletal symptoms [24,27]. Studies have shown that holding the neck in a bent posture and working in the same posture for prolonged periods of time were both significantly associated with neck pain [28]. The findings of the present study suggests that modification of incorrect postures at work and improvements in the ergonomic designs of workstations could be important not only as primary preventive strategies but also as a secondary preventive measure in those with symptoms. However the economic burden to employers, especially in developing countries like Sri Lanka hinders the complete improvements to workstation design. In such instances simple tools such as the OSHA VDT workstation checklist could be effectively utilized by employers in prioritizing issues (Table 3).

Psychosocial factors are also important determinants of CANS among computer office workers. In a systematic review it has been found that high job demand, low decision autonomy, time pressure, mental stress, job dissatisfaction, high workload, and lack of support from colleagues and superiors are risk factors for CANS [10]. The present study evaluated variables of the Karasek model in several domains (job demands, job control, social support and break time). Work overload (speeding to finish tasks on time, finding work tasks difficult and having too many tasks), poor social support (colleagues and superiors) and lack of autonomy (participation in decision making, deciding own task changes and determining time & speed job tasks) were associated with CANS and also determined its' severity. The similarity between odds ratios of the identified psychosocial factors and physical factors might suggest an equal contribution by both in the pathogenesis of CANS. However strategies aimed at modification of psychosocial factors such as social support could be economically more efficient in an employers' perspective.

The study participants also demonstrated relatively poor knowledge and awareness pertaining to ergonomics. In addition in those who were aware of 'Ergonomics', a majority lacked specific knowledge necessary for proper implementation. Poor ergonomic knowledge was also a significant predictor of complaints in the neck, forearm and hand regions. Implementation of a worksite ergonomics programs are known to be effective in reducing work-related complaints in the workforce [29]. In addition, awareness programmes are also known to be cost-effective investments for employers', as it reduces the occurrence of symptoms, improves productivity and reduces medical expense [30]. The other potential barrier to successful primary and secondary prevention of this common problem in Sri Lanka is the relative lack of awareness related to the issue shown by health care professionals. The probable causes could be; the underestimation of the extent and common nature of the problem, lack of awareness on cause-effect relationship and hence non-attribution of symptoms to an occupational cause, ignorance of occupational history and the lack of appreciation of the effect of work place modification on symptoms.

The present study has several limitations. The reporting of complaints may have been biased due to the fact that subjects had to report complaints that occurred in the past 12 months which might have introduced recall bias. In addition the present study is a cross-sectional survey, to imply a causative relationship between CANS and potential risk-factors prospective studies are required.

## Conclusions

The prevalence of work-related CANS among computer office workers in Sri Lanka, a developing, South Asian country is high and comparable to prevalence in developed countries. Work-related physical factors, psychosocial factors and lack of awareness were all important associations of CANS. Hence effective preventive strategies need to address all three areas. Further, studies on different interventional models are required to develop an effective preventive strategy for this relatively common and underestimated problem.

## List of abbreviations

CANS: Complaints of Arm Neck and Shoulders; MUEQ: Maastricht Upper Extremity Questionnaire; OSHA: Occupational Safety and Health Administration; VDT: Visual Display Terminal

## Competing interests

The authors declare that they have no competing interests.

## Authors' contributions

SR, DAL and NJ made substantial contribution to conception and study design. SK and NJ were involved in data collection. YSP and PR were involved in refining the study design, statistical analysis and drafting the manuscript. PK, PR and YSP critically revised the manuscript. All authors read and approved the final manuscript

## References

[B1] Huisstede BM Miedema HS Verhagen AP Koes BW Verhaar JA Multidisciplinary consensus on the terminology and classification of complaints of the arm, neck and/or shoulder Occup Environ Med 2007 64 313 319 10.1136/oem.2005.023861 17043078 PMC2092547

[B2] Gerr F Marcus M Monteilh C Epidemiology of musculoskeletal disorders among computer users: lesson learned from the role of posture and keyboard use J Electromyogr Kinesiol 2004 14 25 31 10.1016/j.jelekin.2003.09.014 14759747

[B3] Klussmann A Gebhardt H Liebers F Rieger MA Musculoskeletal symptoms of the upper extremities and the neck: a cross-sectional study on prevalence and symptom-predicting factors at visual display terminal (VDT) workstations BMC Musculoskelet Disord 2008 9 96 10.1186/1471-2474-9-96 18588677 PMC2474829

[B4] Bongers PM Ijmker S van den Heuvel S Blatter BM Epidemiology of work related neck and upper limb problems: psychosocial and personal risk factors (part I) and effective interventions from a bio behavioural perspective (part II) J Occup Rehabil 2006 16 279 302 16850279 10.1007/s10926-006-9044-1

[B5] Eltayeb SM Staal JB Hassan AA Awad SS de Bie RA Complaints of the arm, neck and shoulder among computer office workers in Sudan: a prevalence study with validation of an Arabic risk factors questionnaire Environ Health 2008 7 33 10.1186/1476-069X-7-33 18588691 PMC2474607

[B6] Ranasinghe P Perera YS Lamabadusuriya DA Kulatunga S Jayawardana N Rajapakse S Katulanda P Work-related complaints of arm, neck and shoulder among computer office workers in an Asian country: prevalence and validation of a risk-factor questionnaire BMC Musculoskelet Disord 2011 12 68 10.1186/1471-2474-12-68 21463513 PMC3080839

[B7] Andersen JH Harhoff M Grimstrup S Vilstrup I Lassen CF Brandt LP Kryger AI Overgaard E Hansen KD Mikkelsen S Computer mouse use predicts acute pain but not prolonged or chronic pain in the neck and shoulder Occup Environ Med 2008 65 126 131 10.1136/oem.2007.033506 17681996

[B8] Gerr F Marcus M Ensor C Kleinbaum D Cohen S Edwards A Gentry E Ortiz DJ Monteilh C A prospective study of computer users: I. Study design and incidence of musculoskeletal symptoms and disorders Am J Ind Med 2002 41 221 235 10.1002/ajim.10066 11920966

[B9] Dellve L Lagerstrom M Hagberg M Work-system risk factors for permanent work disability among home-care workers: a case-control study Int Arch Occup Environ Health 2003 76 216 224 12690496 10.1007/s00420-002-0414-5

[B10] Bongers PM Kremer AM ter Laak J Are psychosocial factors, risk factors for symptoms and signs of the shoulder, elbow, or hand/wrist?: a review of the epidemiological literature Am J Ind Med 2002 41 315 342 10.1002/ajim.10050 12071487

[B11] Department of census and statistics Sri Lanka Census of population and housing 2001, Population, Intercensal growth and average annual rate of growth by district, 1981 and 2001 http://www.statistics.gov.lk/PopHouSat/PDF/Population/p9p1%20Growth.pdf (accessed 10th April 2011)

[B12] Department of census and statistics Sri Lanka: Computer literacy of Sri Lanka 2009 http://www.statistics.gov.lk/CLS/BuletinComputerLiteracy_ 2009.pdf URL (accessed 10th April 2011)

[B13] Eltayeb S Staal JB Kennes J Lamberts PH de Bie RA Prevalence of complaints of arm, neck and shoulder among computer office workers and psychometric evaluation of a risk factor questionnaire BMC Musculoskelet Disord 2007 8 68 10.1186/1471-2474-8-68 17629925 PMC1952062

[B14] Karasek R Brisson C Kawakami N Houtman I Bongers P Amick B The Job Content Questionnaire (JCQ): an instrument for internationally comparative assessments of psychosocial job characteristics J Occup Health Psychol 1998 3 322 355 9805280 10.1037//1076-8998.3.4.322

[B15] Hannan LM Monteilh CP Gerr F Kleinbaum DG Marcus M Job strain and risk of musculoskeletal symptoms among a prospective cohort of occupational computer users Scand J Work Environ Health 2005 31 375 386 16273964 10.5271/sjweh.921

[B16] Snyder LA Krauss AD Chen PY Finlinson S Huang YH Occupational safety: application of the job demand-control-support model Accid Anal Prev 2008 40 1713 1723 10.1016/j.aap.2008.06.008 18760100

[B17] Sanne B Mykletun A Dahl AA Moen BE Tell GS Testing the job demand-control-support model with anxiety and depression as outcomes: the Hordaland health study Occup Med 2005 55 463 473 10.1093/occmed/kqi071 15845554

[B18] Government of Sri Lanka Provincial Councils of Sri Lanka 2005 http://www.priu.gov.lk/ProvCouncils/Indexpc.html (accessed 17th April 2011)

[B19] Computer Workstation eTool Occupational Safety and Health Administration Web site http://www.osha.gov/SLTC/etools/computerworkstations/index.html Available from: (accessed 12th April 2011)

[B20] Bekiari EI Lyrakos GN Damigos D Mavreas V Chanopoulos K Dimoliatis ID A validation study and psychometrical evaluation of the Maastricht Upper Extremity Questionnaire (MUEQ) for the Greek-speaking population J Musculoskelet Neuronal Interact 2011 11 52 76 21364275

[B21] Sillanpää J Huikko S Nyberg M Kivi P Laippala P Uitti J Effect of work with visual display units on musculo-skeletal disorders in the office environment Occup Med (Lond) 2003 53 443 451 10.1093/occmed/kqg120 14581641

[B22] Juul-Kristensen B Jensen C Self-reported workplace related ergonomic conditions as prognostic factors for musculoskeletal symptoms: the ''BIT'' follow up study on office workers Occup Environ Med 2005 62 188 194 10.1136/oem.2004.013920 15723884 PMC1740969

[B23] Marcus M Gerr F Monteilh C Ortiz DJ Gentry E Cohen S Edwards A Ensor C Kleinbaum D A prospective study of computer users: II. Postural risk factors for musculoskeletal symptoms and disorders Am J Ind Med 2002 41 236 249 10.1002/ajim.10067 11920967

[B24] IJmker S Huysmans MA Blatter BM van der Beek AJ van Mechelen W Bongers PM Should office workers spend fewer hours at their computer? A systematic review of the literature Occup Environ Med 2007 64 211 222 17095550 10.1136/oem.2006.026468 PMC2078461

[B25] Nakazawa T Okubo Y Suwazono Y Kobayashi E Komine S Kato N Nogawa K Association between duration of daily VDT use and subjective symptoms Am J Ind Med 2002 242 421 426 10.1002/ajim.10133 12382255

[B26] Skov T Borg V Orhede E Psychosocial and physical risk factors for musculoskeletal disorders of the neck, shoulders, and lower back in salespeople Occup Environ Med 1996 53 351 356 10.1136/oem.53.5.351 8673184 PMC1128479

[B27] Gerr F Monteilh CP Marcus M Keyboard use and musculoskeletal outcomes among computer users J Occup Rehabil 2006 16 265 277 16802184 10.1007/s10926-006-9037-0

[B28] Ariens GA Bongers PM Hoogendoorn WE van der Wal G van Mechelen W High physical and psychosocial load at work and sickness absence due to neck pain Scand J Work Environ Health 2002 28 222 31 12199423 10.5271/sjweh.669

[B29] Cole DC Hogg-Johnson S Manno M Ibrahim S Wells RP Ferrier SE Worksite Upper Extremity Research Group Reducing musculoskeletal burden through ergonomic program implementation in a large newspaper Int Arch Occup Environ Health 2006 80 98 108 10.1007/s00420-006-0107-6 16736193

[B30] Hendrik HW Human Factors and Ergonomics Society The ergonomics of economics in the economics of ergonomics Proceedings of the 40th Annual Meeting of the Human Factors and Ergonomics Society: 2-6 September 1996; Santa Monica 1996 1 10

